# Maternal suboptimal selenium intake and low-level lead exposure affect offspring’s microglial immune profile and its reactivity to a subsequent inflammatory hit

**DOI:** 10.1038/s41598-023-45613-2

**Published:** 2023-12-05

**Authors:** R. De Simone, M. A. Ajmone-Cat, A. M. Tartaglione, G. Calamandrei, L. Minghetti

**Affiliations:** 1 National Center for Drug Research and Evaluation, Istituto Superiore di Sanità, 00161 Rome, Italy; 2 Center for Behavioral Sciences and Mental Health, Istituto Superiore di Sanità, 00161 Rome, Italy; 3 Research Coordination and Support Service, Istituto Superiore di Sanità, 00161 Rome, Italy

**Keywords:** Cell biology, Molecular biology, Neuroscience

## Abstract

Micronutrients such as selenium (Se) are essentials since prenatal life to support brain and cognitive development. Se deficiency, which affects up to 1 billion people worldwide, can interact with common adverse environmental challenges including (Pb), exacerbating their toxic effects. Exploiting our recently validated rat model of maternal Se restriction and developmental low Pb exposure, our aims were to investigate: (i) the early consequences of suboptimal Se intake and low-Pb exposure on neuroinflammation in neonates’ whole brains; (ii) the potential priming effect of suboptimal Se and low-Pb exposure on offspring’s glial reactivity to a further inflammatory hit. To these aims female rats were fed with suboptimal (0.04 mg/kg; Subopt) and optimal (0.15 mg/kg; Opt) Se dietary levels throughout pregnancy and lactation and exposed or not to environmentally relevant Pb dose in drinking water (12.5 µg/mL) since 4 weeks pre-mating. We found an overall higher basal expression of inflammatory markers in neonatal brains, as well as in purified microglia and organotypic hippocampal slice cultures, from the Subopt Se offspring. Subopt/Pb cultures were highly activated than Subopt cultures and showed a higher susceptibility to the inflammatory challenge lipopolysaccharide than cultures from the Opt groups. We demonstrate that even a mild Se deficiency and low-Pb exposure during brain development can influence the neuroinflammatory tone of microglia, exacerbate the toxic effects of Pb and prime microglial reactivity to subsequent inflammatory stimuli. These neuroinflammatory changes may be responsible, at least in part, for adverse neurodevelopmental outcomes.

## Introduction

Early life exposure to environmental factors, including nutrition, can profoundly affect an individual’s phenotype and its predisposition to develop diseases during infancy as well as at adulthood^1–4^. Among the essential dietary elements required for normal growth and development are micronutrients, such as selenium (Se), a trace element of great importance for animal and human health^5^.

Recommendations for Se intake average 60–70 µg per day for adults; the EFSA set the Adequate Intake at 70 µg per day for adults of both sexes and 85 µg per day for lactating women^6^.

Research in animal models and human populations has shown that maternal Se deficiency can increase the risk of a range of pregnancy complications and is associated with adverse disease outcomes later in life^7,8^. Se effects are mainly exerted through selenoproteins, a class of proteins that incorporate Se co-translationally in the form of selenocysteine, which play pivotal roles in the antioxidant defence system, thyroid hormone metabolism, fertility, and innate and adaptive immune responses^9–11^. In addition to these well-documented functions, Se plays an essential role in brain development and function, as revealed by a number of experimental and epidemiological studies (see^12,13^ and refs therein). Se deficiency in mothers is directly reflected in a poor Se status in the newborns^14^ and influences children's psychomotor, language, and cognitive development^15–17^. Se deficiency affects up to 1 billion people worldwide^18^, due to inadequate intake, and Se deficiency risk has been predicted to increase under changes in climate and soil organic carbon content, particularly in agricultural areas^19^.

Very low selenium concentration is associated with numerous diseases, such as endemic osteoarthropathy (Kashin–Beck disease) and dilated cardiomyopathy (Keshan disease)^20^, cardiovascular disease, infertility, myodegenerative diseases, and cognitive decline (see^18^). It is important to note that even a mild Se deficiency can have high impact on human health when in the presence of adverse environmental factors, such as pollutants and food contaminants, especially in sensitive developmental windows. Notably, in previous developmental studies in rats, we demonstrated that a moderate maternal Se deficiency—due to suboptimal dietary intake (0.04 mg/kg Se content as l-selenomethionine, the main Se chemical species in the diet)—has significant effects on neurobehavioral development and expression of inflammatory/plasticity-related genes in both cortex and hippocampus at juvenile stages^21^. In addition, we provided evidence of the potential interaction of the maternal Se status with lead (Pb), a common adverse environmental challenge and a growing public health concern worldwide. Although acute lead poisoning has become rare in developed countries, chronic exposure to low levels of the metal commonly occurs through occupational and environmental sources, through inhalation or ingestion of Pb-contaminated dust, water, and food and from hand-to-mouth behavior. Pb exposure can permanently impair different aspects of human health, and affect the proper development of various vital systems, including the nervous system, causing cognitive dysfunction, neurobehavioral disorders, and neurological damage^22–26^. No safe blood lead level (BLL) in children has been identified, and 3.5 µg/dL has been proposed as blood Pb reference value for children by Center for Disease Control and Prevention (https://www.cdc.gov/nceh/lead/news/cdc-updates-blood-lead-reference-value.html),^22–28^. A benchmark dose lower confidence limit (BMDL) of 1% extra risk equal to 1.2 μg Pb/dL has been identified by the EFSA as a reference point for the risk characterization of Pb when assessing the risk of intellectual deficits in children up to the age of 7 years measured by the Full-Scale IQ score^29^.

Our previous rodent study demonstrated that the co-occurrence of maternal suboptimal Se status with developmental low-level Pb exposure worsened Pb adverse effects on selected neurobehavioral aspects at early and later stages of development, as well as on the developing juvenile glutamatergic system in the hippocampus^30^. The dose of Pb used (12.5 µg/mL in drinking water) is among the lowest used in rodent studies and originates blood Pb levels in the rat offspring corresponding to those produced by environmental “sub-toxic” relevant exposure in children^31^, without inducing overt reproductive or systemic toxicity neither alone nor in combination with suboptimal Se in the maternal diet. We therefore used the same dose in the present study to closely mimic a real scenario of chronic low exposure since early life.

Epidemiological studies on Se status in pregnancy and combined early exposure to chemical stressors such as Pb in large birth cohort studies are few, despite the well-documented detrimental effects of inadequate dietary Se intake and Pb exposure per se in early life. A recent prospective birth cohort study on the long-term influences of Se deficiency with prenatal Pb exposure on the neuropsychological outcome of school-age children^32^ evidenced the shielding effect of optimal maternal dietary Se intake against the negative impact of Pb on child cognitive abilities, at Pb concentrations even lower than the threshold considered as overtly neurotoxic by regulatory agencies.

Although the possible protective role of Se against developmental Pb exposure-related neurotoxicity in humans remains to be conclusively demonstrated (see for example^33^), our study as well as the experimental evidence of the potential benefit of Se supplements against Pb neurotoxicity^23,34,35^ highlight the importance of further exploring the interaction between this micronutrient and Pb in critical brain developmental windows.

Maternal nutritional deficiencies, and/or perinatal exposure to toxicants can be regarded as a first hit that “programs” the offspring for increased susceptibility to further metabolic, behavioral, and inflammatory hits later in life^4,36,37^. Long-lasting alterations in the properties of microglia, the brain resident macrophages, are suggested to account at least in part for these outcomes^38,39^, being these cells critically important for neural circuitry establishment during brain development. By acquiring distinct phenotypes with unique gene expression profiles during the different phases of development, microglia shape neuronal circuits and regulate neurogenesis, actively contributing to functional synapse formation and maturation^40^. Experimental evidence indicates that these cells can be particularly sensitive to the effects of Se and Pb. The knockdown of mSELENOK (one of the selenoproteins by which Se acts) impairs the migration and phagocytosis of microglia^41^, and several studies had shown the anti-inflammatory effects of Se in vivo and in vitro (for a review see^42^). As concerning Pb, a large body of evidence indicated that exposure to this heavy metal may result in microgliosis and inflammation (for a review see^43^). This knowledge prompted us to focus on microglia as one of the most likely key cell targets in the maternal programming of offspring’s brain in conditions of inadequate Se intake and Pb exposure.

The objectives of the current study were twofold: first, to determine the effects of a prolonged Suboptimal low maternal Se intake on the brain neuroinflammatory profile of the progeny at birth and infancy; second, to test the hypothesis that low maternal Se intake would exacerbate the effects of perinatal Pb exposure and prime microglia to enhanced reactivity to a further inflammatory stimulus in the postnatal period.

To these aims, we used our recently validated in vivo model of Se restriction and low-Pb exposure (i) to analyse whole brains from the offspring at birth for their expression of inflammatory markers and (ii) to establish purified cortical microglial cultures and organotypic hippocampal slice cultures (OHSCs) from postnatal day (PND) 1 and PND 6 pups, respectively, assessing their state of activation and reactivity to the typical inflammatory agent lipopolysaccharide (LPS).

Our data support the notion that adequate Se intake during gestation has a protective role on brain immune properties against the toxic effects of Pb exposure and against further challenges later in life, underlining the importance of adequate Se intake since early developmental phases.

## Methods

### Animals and experimental design

This study is reported in accordance with ARRIVE. Wistar rats were kept under standard animal housing (temperature 20 ± 2 °C; humidity 60–70%) with food and water ad libitum, under a 12 h-12 h light/dark cycle (lights on from 7:00 a.m. till 7:00 p.m.). Following the adaption period and 8 weeks prior to breeding, females (weighing 250 ± 25 g) were randomly allocated to either an optimal or suboptimal dietary Se intake group (Se Opt or Se Subopt, respectively; see “Se-containing diets”). After the first 4 weeks under this dietary regimen, female rats from both the Se Opt and Se Subopt groups were further randomly divided into two subgroups based on exposure to Pb or Vehicle (Veh). This ensured that half of the females in each group received water containing 12.5 µg/mL of Pb in their drinking bottles, while the other half received water with no Pb (Veh) throughout pregnancy and lactation until the days of sacrifice (PND 1 or 6). Thus, the resulting four experimental groups were: Se Opt/Veh, Se Opt/Pb, Se Subopt/Veh, and Se Subopt/Pb.

**Figure 1:**
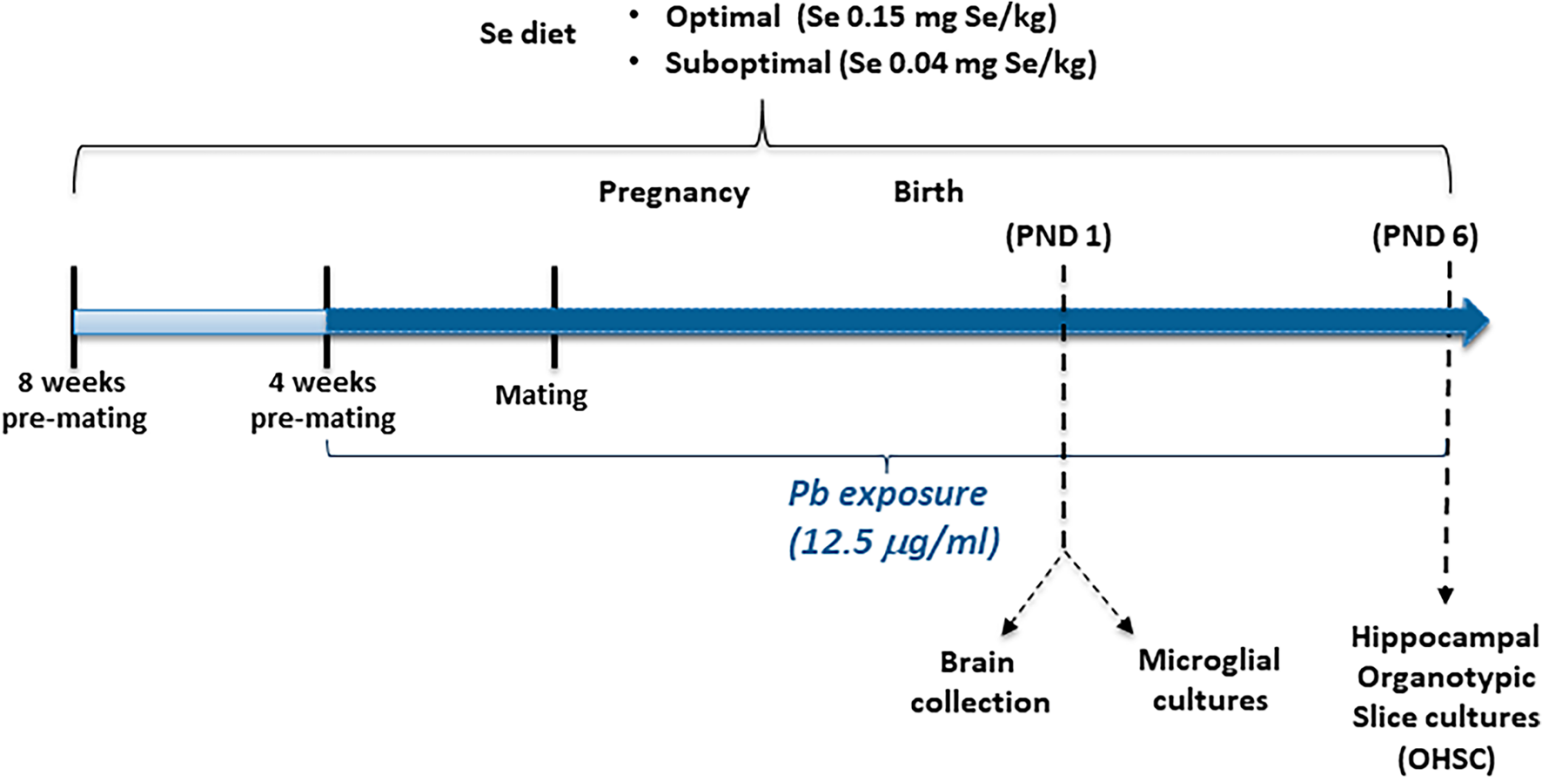
Depiction of experimental groups and study design. Se: selenium; Pb: Lead acetate (Pb(Ac)_2_·3H_2_O); PND: postnatal day.

Female rats were mated with males (2:1) for 4 to 5 days to cover the duration of an estrous cycle. The animals were regularly weighed, and their food and water consumption recorded every other day. The timeline of experimental design is reported in Fig. 1.

### Se-containing diets

Rat diets were prepared by Envigo by supplementing a selenium deficient diet (Teklad Custom Diet TD.92163), containing < 0.01 mg Se/kg with l-selenomethionine to obtain a diet with optimal (Opt, 0.15 mg Se/kg) or suboptimal (Subopt, 0.04 Se mg/kg) Se content, respectively.

### Pb exposure

Pb acetate (Pb(Ac)_2_·3H_2_O) was purchased from Sigma Aldrich (Merck KGaA, Darmstadt, Germany). Test solutions for animal treatment were prepared in soft tap water, slightly acidified (pH 4.6) with acetic acid, and containing no Pb acetate (Veh) or Pb acetate at a concentration 12.5 µg/mL of Pb (as element). The selected medium ensured complete dissolution of the Pb salt and acceptance by the animals was the same as plain tap water. The Pb concentration in the test solutions was analytically checked and stability was verified for 5 days. The solutions were prepared fresh every 3–4 days and used as drinking water for animal treatment. The resulting exposure was ~ 1.25 mg/kg body weight Pb per day.

### Whole brain mRNA extraction

The day of birth was designated as PND 0. At PND 1, 6 pups from 6 different litters/group were sacrificed by decapitation under anesthesia induced by hypothermia. and whole brains rapidly dissected on ice; olfactory bulb and medulla were discarded, and the rest of the tissue was snap frozen and kept at − 80 °C until RNA extraction with Trizol reagent.

### Purified microglial culture preparation and stimulation

Each mixed primary glial culture was obtained from the cerebral cortex of 1 rat pup/litter from 4 different litters/group (at PND 1), sacrificed as above. following previously published procedures^44^. Entire removal of meninges ensured negligible blood contaminants. Mixed primary glial cultures were maintained in Basal Eagle's Medium (BME), supplemented with 10% heat‐inactivated foetal bovine serum, 2 mM glutamine and 100 μg/mL gentamicin. Medium was replaced after 3 days and cultures maintained in the same medium as above for additional 8 days. After this time, microglial cells were harvested by mild shaking, resuspended in fresh medium without serum, and seeded on uncoated plastic wells at the density of 1 × 10^5^cells/cm^2^. Cell viability was > 95% in all culture conditions, as tested by Trypan Blue exclusion. The cultures consisted of 96% positive cells for the microglia/macrophage marker CD68 (ED-1; 1:200; Serotec). After 24 h in culture, the cells were stimulated or not with 10 ng/ml lipopolysaccharide (LPS, Sigma), in fresh medium. Following 24 h of incubation, the culture media were collected, centrifuged at 1500 rpm to remove cell debris, and stored at − 20 °C until biochemical analyses. Cell monolayers were harvested in Trizol reagent and stored at − 80 °C until RNA extraction.

### Organotypic hippocampal slice cultures (OHSC)

Organotypic hippocampal slice cultures (OHSC) were prepared as described in^45^, with few modifications. In brief, 6 rat pups from 6 different litters/group were decapitated (PND 6), and brains rapidly dissected and placed in ice-cold dissection medium (HBSS 1x; HEPES 20 mM; Pen/Strep100 U/ml; d-glucose 6 mg/ml); the hippocampi were isolated and sectioned into 350-μm transverse slices with a McIlwain tissue chopper (Mickle Laboratory Engineering Co. Ltd, Goose Green, UK). The slices were then carefully separated and transferred onto porous collagenated membrane inserts (four slices per insert; PICMORG50, Millicell, Millipore), in 6-well culture plates. 1200 μL culture medium, consisting of Neurobasal^A^ medium, 1 mM l-glutamine, 100 U/mL penicillin and 100 µg/mL streptomycin, were added to the lower compartment of each well. On the next day of culture, the culture media were replaced with fresh medium and, from that time, changed twice a week. The slices were incubated at 35 °C, 5% CO_2_ for 7 days, before stimulation or not with LPS (5 μg/ml; Sigma), to allow the inflammatory reaction following the mechanical procedure to subside. OHSCs viability was routinely assessed by visualization of propidium iodide (PI; 5 μg/ml; Sigma) staining and fluorescence microscopy; not vital slices were discarded. Following 24 h of LPS stimulation culture media were collected as described above, and slices transferred in Trizol for RNA extraction. Four pups per experimental group were used in these experiments.

### RNA extraction, reverse transcription, and real-time PCR analyses

For gene expression experiments, RNA was extracted using Trizol reagent. The quality and concentration of RNA were assessed at NanoDrop spectrophotometer (Thermo Scientific). cDNA was reverse transcribed from 1 µg of RNA using the High-Capacity cDNA Reverse Transcription Kit (Applied Biosystems, Thermo Fisher Scientific). Real-time PCR was performed with a 7500 Real-time PCR System apparatus (Applied Biosystems, Thermo Fisher Scientific) on the reverse transcription products with TaqMan master mix and TaqMan™Gene Expression Assays (Applied Biosystems, Thermo Fisher Scientific).

Annealing temperature was 60 °C for all the primer pairs listed. All samples were run in duplicate, and each PCR well contained 20 μl as a final volume of reaction, including 2 μl of complementary DNA corresponding to approximately 60 ng total RNA, 750 nM of each primer, and 10 μl PCR master mix. Thermal cycling conditions were as follows: 1 cycle at 95 °C for 10 min; 40 cycles at 95 °C for 15 s, and 60 °C for 1 min. The relative expression level of each mRNA in different experimental groups was calculated using the 2^−ΔΔCt^ method, normalized to HPRT and expressed as fold change relative to the Opt Se group, taken as 1.

### ELISA determination of cytokine levels

IL-1β and IL-6 protein levels accumulated in culture supernatants were assessed by specific ELISAs from Diaclone (detection limit for IL-1β: 4 pg/ml and for IL-6: 12 pg/ml), according to manufacturer’s instructions.

### Nitrite levels determination

Production of nitric oxide (NO) was determined by measuring the content of nitrite, one of the end products of NO oxidation in the media, as previously described^45^. A standard nitrite curve (0.25–50 µM) was generated using a 10 mM solution of NaNO_2_. All chemicals for the NO assay were from Sigma.

### Statistical analyses

Data are expressed as means ± standard error of the mean (SEM). Statistical analyses were performed using unpaired Student’s T-test for comparison between two groups, and Two-way ANOVA for multiple comparisons, where the effect of "diet" is defined as Se diet and "treatment" is defined as Pb exposure. Tukey’s post hoc test on significant interactions was used by employing Prism GraphPad 9.4.1 software. The statistical unit of measure for each endpoint was the litter or individual pup within the litter. *p* values < 0.05 were considered as significant.

### Ethics approval and consent to participate

The study was conducted according to the European and Italian legislation (2010/63/EU, Dl 26/2014), and approved by the Animal Welfare Committee of Istituto Superiore di Sanità and by the Italian Ministry of Health (authorization n◦ 843/2016-PR).

## Results

### Effects of maternal Suboptimal Se diet on brain immune-related gene expression in the offspring

We evaluated whether a prolonged maternal Subopt dietary intake of Se could alter—in their offspring- the basal Central Nervous System (CNS) expression of a selected group of markers involved in immune functions, brain homeostasis and development. Brains were collected on PND 1 from offspring of dams fed with Opt or Subopt Se diets, and the expression of the inflammatory cytokines interleukin (IL)-6 and IL-1β, the immunomodulatory cytokine IL-10, the inflammatory/oxidative stress-related enzymes inducible nitric oxide synthase (iNOS) and arginase-1 (Arg-1), and the microglial/macrophage phagocytic receptors cluster of differentiation (CD)11b and Mannose Receptor C-Type 1 (MRC-1), were analyzed by Real Time PCR.

**Figure 2:**
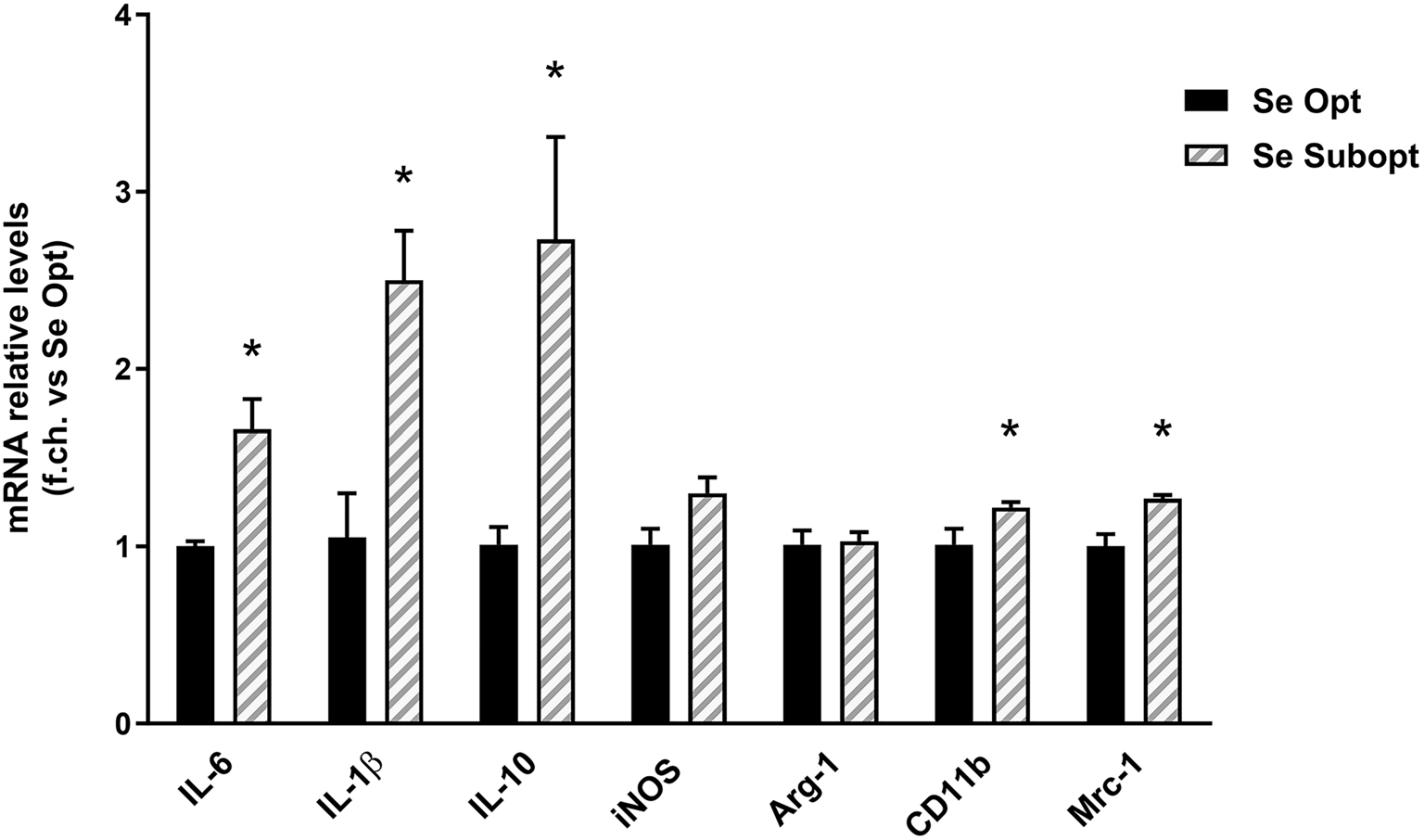
Relative levels of inflammatory gene mRNAs, as measured by real-time RT-PCR in whole brain extracts (PND 1), expressed as fold change versus the levels found in Se Opt group taken as 1, and normalized to HPRT as the reference gene in each sample. The transcript levels for IL-1β, IL-6, IL-10, CD11b and MRC-1 were significantly higher in brains from the Se Subopt group when compared to the Se Opt group. Data are represented as mean ± SEM (n = 6 per group; unpaired T test: *p < 0.05 vs. Se Opt).

We found increased mRNA levels of IL-6, IL-1β, IL-10, CD11b and MRC-1 in the Subopt group when compared to the Opt group (Fig. 2), suggesting that Subopt Se intake during gestation profoundly alters immune/plasticity-related functions in the offspring brain. The levels of the inducible enzyme iNOS, responsible for nitric oxide (NO) production under inflammatory conditions, showed only a tendency to increase in the Subopt group, while Arg-1 levels did not change.

### Effects of different maternal Se dietary intakes and Pb exposure on offspring’s microglia

**Figure 3:**
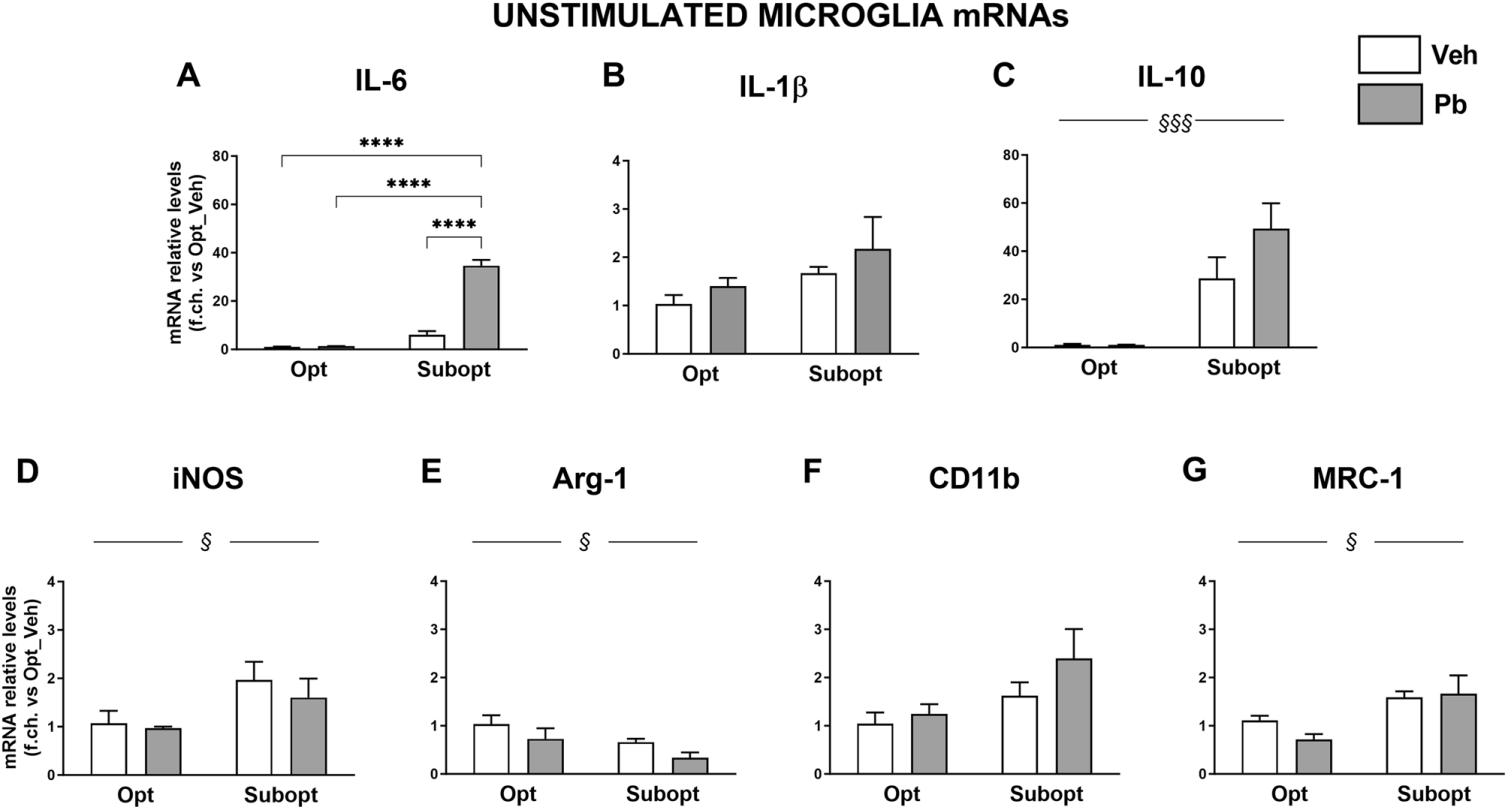
Relative levels of inflammatory gene mRNAs in microglial cells from the cortex of rat pups (PND 1) born to Opt and Subopt fed dams exposed to Pb or Veh (drinking water) during gestation, as measured by real-time RT-PCR. Data are expressed as the fold change versus the level found in Opt/Veh microglia taken as 1, and normalized to HPRT as the reference gene in each sample. (**C**–**E**,**G**) IL-10, iNOS, and MRC-1 expressions were increased in Subopt microglia, while Arg-1 was down-regulated, compared to Opt microglia [main diet effect: F (1,8) = 30.6 ^$$$^*p* = 0.0006 for IL-10; F (1,9) = 5.426 ^$^*p* = 0.0448 for iNOS; F (1,10) = 8.488 ^$^*p* = 0.0155 for MRC-1; F (1,10) = 7.153 ^$^*p* = 0.0233 for Arg-1]. IL-1β (**B**) and CD11b (**F**) showed only a tendency to increase in Subopt microglia compared to Opt [main diet effect: F (1, 9) = 2.488 *p* = 0.1492 for IL1β; F (1, 10) = 4.305 *p* = 0.0648 for CD11b]. Pb did not affect the expression of the genes analyzed, with the exception of IL-6 (**A**) that was upregulated in Subopt condition [interaction diet x Pb: F(1,8) = 95.86 *p* < 0.0001]. Data in A-G are mean ± SEM; n = 4 per group. ****p < 0.0001 (Tukey’s post hoc analyses).

The effects of low Se, Pb exposure and their combination on the properties of neonatal microglia were assessed in microglial cultures from the cortex of rat pups (PND 1) from each of the four experimental groups i.e.: Se Opt/Veh, Se Opt/Pb, Se Subopt/Veh, and Se Subopt/Pb, analysing the mRNA expression of the inflammatory markers examined in whole brain extracts (i.e. IL-6, IL-1β, IL-10, iNOS, Arg-1, MRC-1, CD11b; Fig. 3).

IL-10, iNOS, and MRC-1 mRNAs levels (Fig. 3C,D,G, respectively) were higher in microglia from Subopt compared to the Opt group, in line with what observed in whole brain extracts, while IL-1β (Fig. 3B) and CD11b (Fig. 3F) showed only a tendency to increase. In addition, Arg-1 (Fig. 3E) was down-regulated in Subopt microglia. Exposure to Pb during gestation did not further modify the specific expression of these genes in offspring’s microglia due to diet (Opt or Supopt Se-diet), whereas it strongly increased that of IL-6 (Fig. 3A) in conditions of Subopt Se intake only (2-way ANOVA results and Tukey’s post hoc analyses for all genes are reported in Fig. 3 legend).

### Effects of different maternal Se dietary intakes and Pb exposure on offspring’s microglial reactivity to LPS

**Figure 4:**
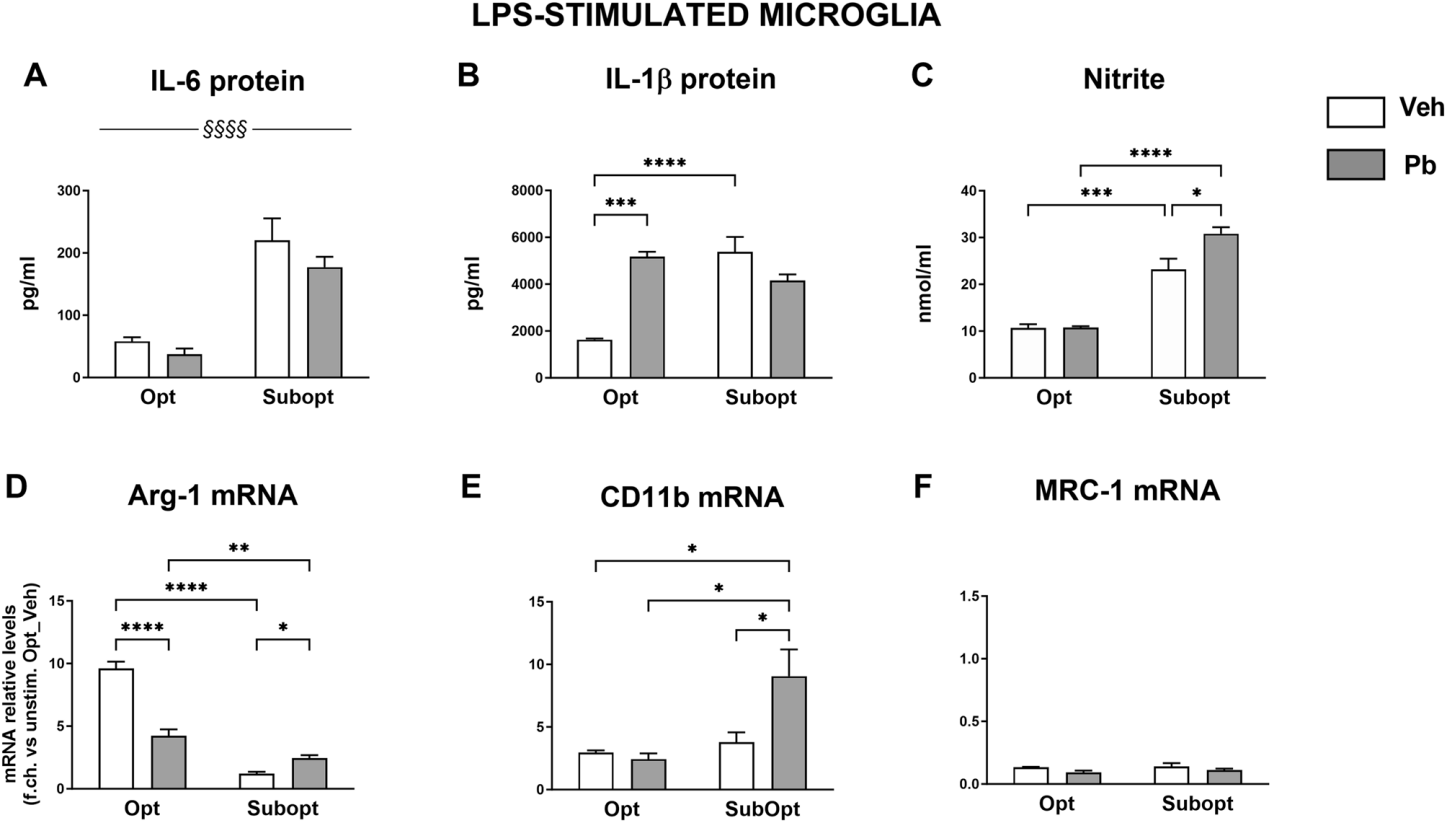
Inflammatory marker regulation in LPS-stimulated cortical microglia. Metabolite levels were measured in cell supernatants by cytokine-specific ELISAs and Griess reaction for nitrite quantitation (**A**–**C**), while relative transcript levels were measured by real-time RT-PCR on cell extracts (**D**–**F**). (**A**–**C**): A main effect of diet was found for IL-6 [F (1,14) = 38.59 ^§§§§^*p* < 0.0001], with Se Subopt cultures bearing the highest protein levels. Developmental Pb exposure enhanced nitrite and IL-1β production in a dietary Se-dependent manner [diet x Pb interaction: F (1,9) = 7.831 *p* = 0.0208 for nitrite; F (1,14) = 31.3 *p* < 0.0001 for IL-1β]. (**D**–**F**): A significant interaction diet x Pb was found for Arg-1 mRNA [F (1, 13) = 102.1 *p* < 0.0001], and for CD11b mRNA [F (1, 10) = 6.794 *p* = 0.0262]. MRC-1 mRNA levels were comparable in all groups. Data in (**A**–**F**) are the mean ± SEM; n = 4 per group. Tukey’s post hoc analyses: *p < 0.05, **p < 0.005, ***p < 0.0005, ****p < 0.00005.

To test whether Suboptimal maternal Se intake and Pb exposure could alter offspring microglia reactivity to a further inflammatory challenge, microglial cultures isolated from the cortex of PND1 rat pups from the four experimental groups as above, were stimulated with 10 ng/ml LPS for 24 h. At the end of treatment, we measured the levels of nitrite (as an index of NO production) and the accumulation of the cytokines IL-1β and IL-6, in microglial supernatants. As expected, after LPS stimulation, the levels of these metabolites were significantly induced in all experimental groups (Fig. 4A–C), while they remained undetectable in unstimulated cultures (not shown).

Higher levels of IL-6, IL-1β and nitrite, as well as reduced levels of Arg-1 were measured in Subopt/Veh compared to Opt/Veh cultures (Fig. 4A–D), suggesting a greater reactivity to LPS in suboptimal Se condition. The co-occurrence of Pb exposure did not modify IL-6 production while we found a strong interaction between diet and Pb in the modulation of IL-1β and nitrite. Indeed, perinatal Pb exposure enhanced nitrite production in response to LPS in Subopt cultures, while increased that of IL-1β only in Opt microglia. 2-way ANOVA results and Tukey’s post hoc analyses for all metabolites are reported in Fig. 4 legend.

In the cell extracts obtained from the same cultures, we analyzed the mRNA expression of the enzyme Arg-1, and of the receptors CD11b and MRC-1 (Fig. 4D,E). As expected, LPS upregulated Arg-1 and CD11b expression in all experimental groups, while it reduced that of MRC-1. Also in this case, the effects of perinatal Pb exposure were dependent on maternal Se intake, as Arg-1 mRNA levels were reduced by Pb in condition of optimal Se intake and moderately increased in suboptimal condition. In any case, as a net effect, Arg-1 levels in Subopt/Pb cultures were lower than in Opt/Pb. As concerning CD11b, Pb exposure had no effect in optimal Se conditions while it further enhanced its expression in Subopt cultures.

At variance with the other genes analyzed, MRC-1 mRNA levels (Fig. 4F) were comparable in all four experimental groups and remained lower than in control-unstimulated cultures (2-way ANOVA and Tukey’s post hoc values for all the genes analyzed are reported in Fig. 4 legend).

### Effects of different maternal Se dietary intakes and Pb exposure on hippocampal immune homeostasis and reactivity to LPS in the offspring: organotypic hippocampal slice cultures (OHSCs)

**Figure 5:**
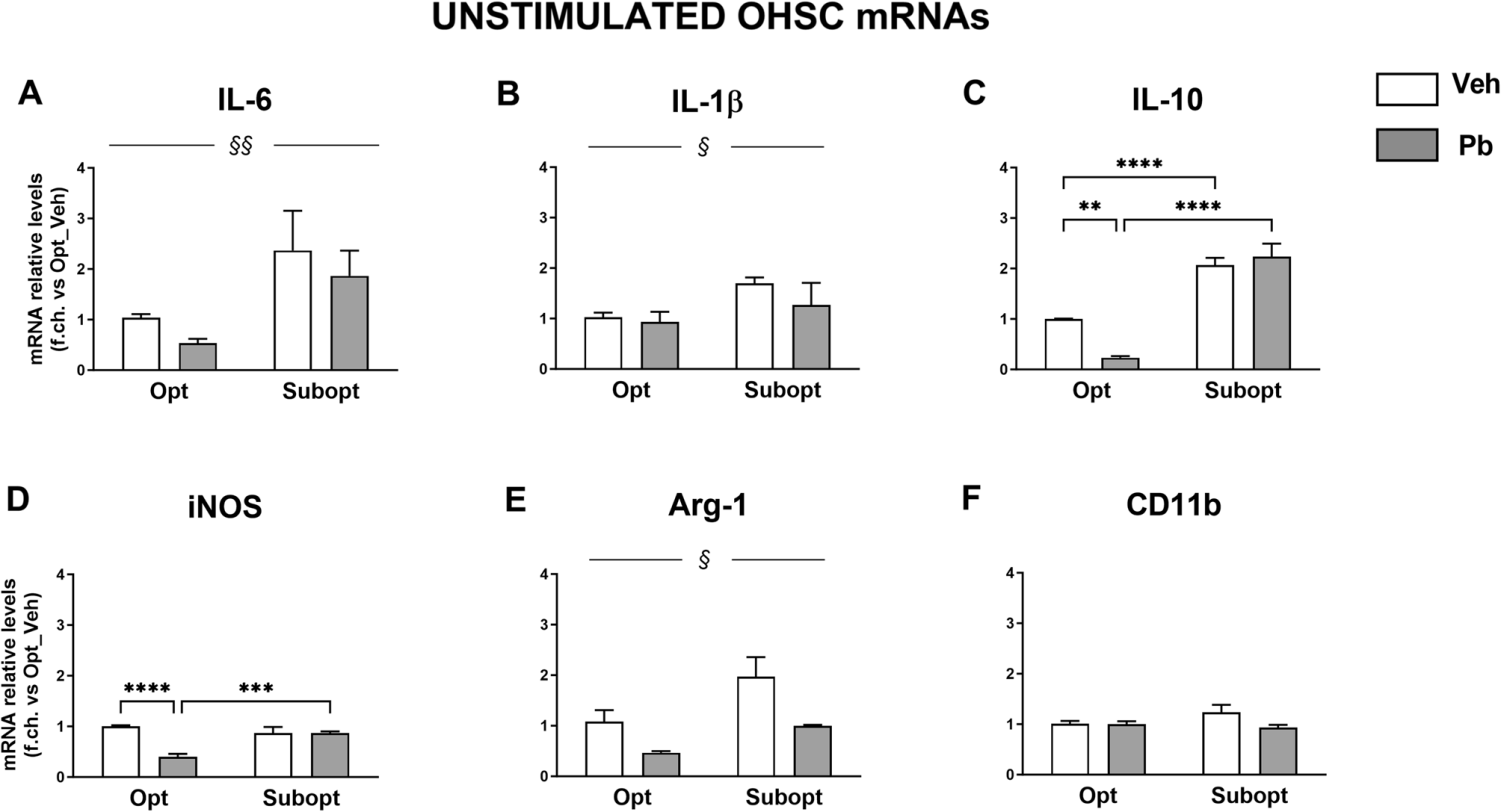
Basal gene expression of inflammatory markers in unstimulated OHSCs, measured by real-time RT-PCR. Data are expressed as the fold change versus the levels found in Se Opt/Veh group taken as 1 and normalized to HPRT as the reference gene in each sample. (**A**–**C**,**E**) Higher mRNA levels of IL-6, IL-1β, IL-10 and Arg-1 were expressed in Se Subopt- vs. Opt-derived OHSCs [main diet effect: F (1, 10) = 11.10 ^§§^*p* = 0.0076 for IL-6; F (1, 12) = 5.800 ^§^*p* < 0.033 for IL-1β; F (1, 12) = 181.8 *p* < 0.0001 for IL-10; F (1, 10) = 8,516; *p* = 0.0153 for Arg-1]. Pb exposure down-regulated Arg-1 (**E**) independently from the diet [treatment main effect: F (1, 10) = 10,64 *p* = 0,0086], while reducing IL-10 (**C**) and iNOS (**D**) mRNAs only in cultures from Se Opt fed rats [diet x Pb interaction: F (1, 12) = 16.84 *p* = 0.0015 for IL-10; F (1, 12) = 28,35 *p* = 0.0002 for iNOS]. Tukey’s post hoc analyses in (**A**–**F**): **p < 0.005, ***p < 0.0005, ****p < 0.00005. Mean ± SEM; n = 4 per group.

To assess the possible enduring consequences of different dietary Se contents and Pb exposure throughout pregnancy and lactation on hippocampal immune homeostasis in the offspring, we established OHSCs from PND 6 pups. To avoid the confounding effects of the inflammatory reaction consequent to the mechanical manipulation of the slices, OHSCs were cultured for 1 additional week before stimulation. The slices were then either stimulated or not with LPS (5 µg/ml) for 24 h, and the mRNA levels of IL-6, IL-1β, IL-10, iNOS, Arg-1 and CD11b, were evaluated by Real Time PCR (Fig. 5).

As shown in Fig. 5A–C,E, basal OHSCs obtained from the Se Subopt groups had higher mRNA levels of IL-6, IL-1β, IL-10, consistently with a state of higher activation, as observed in whole neonatal brains and neonatal purified cortical microglial cultures. Arg-1 mRNA was upregulated, differently from what observed in whole brain extracts and cortical microglia, suggesting a brain-region specific regulation of this gene.

Perinatal Pb exposure did not change the expression of IL-6 and IL-1β whereas it reduced that of Arg-1, independently from the diet. A strong interaction between diet and Pb was found for IL-10 and iNOS mRNAs (Fig. 5C,D) that were downregulated only in optimal Se condition (2-way ANOVA and Tukey’s post hoc results are reported in the figure legend). As for CD11b mRNA levels (Fig. 5F), they were comparable in all groups.

**Figure 6:**
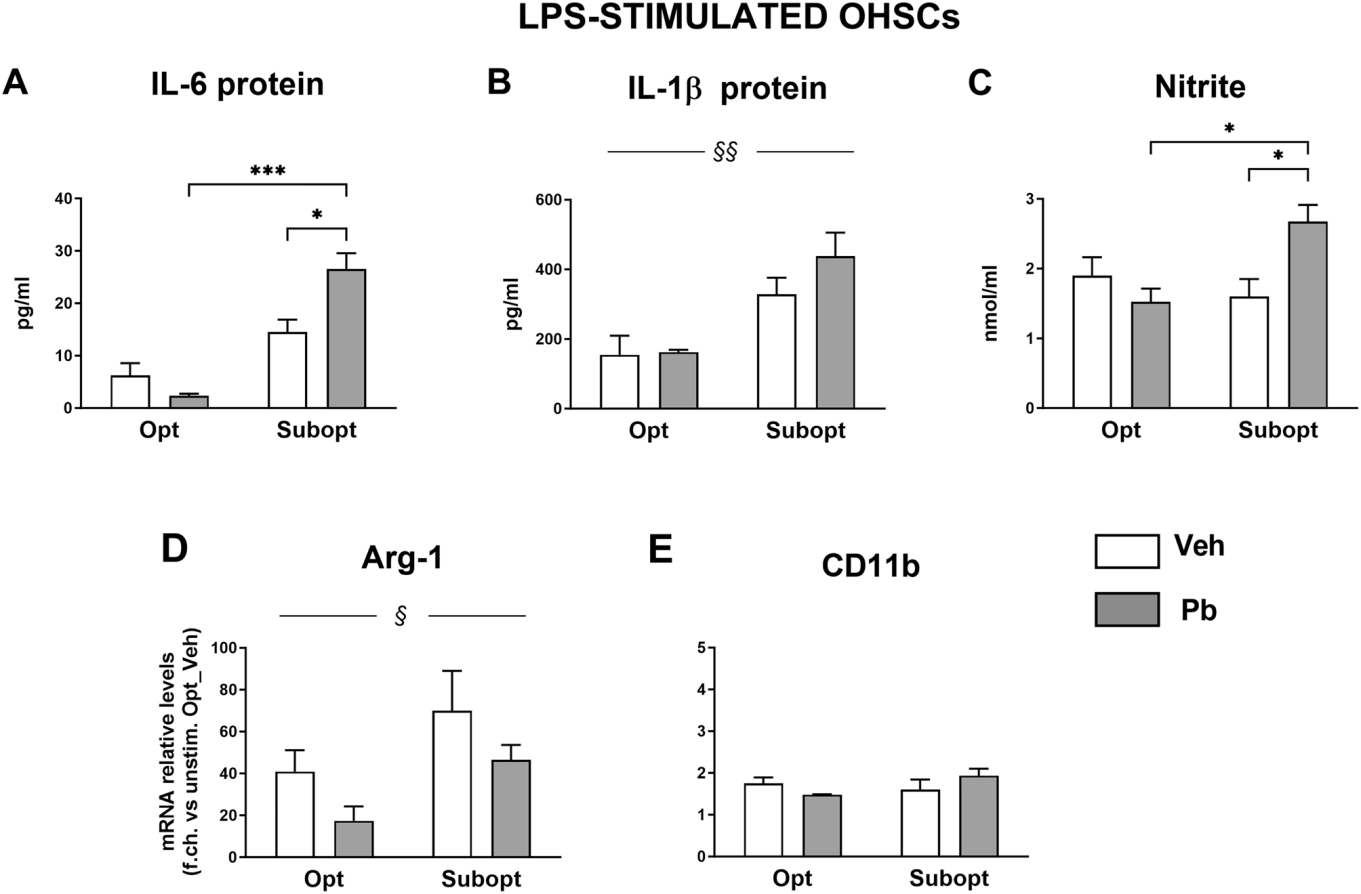
Inflammatory marker regulation in LPS-stimulated organotypic hippocampal slices (OHSCs). Metabolites levels were measured in cell supernatants by cytokine-specific ELISAs and Griess reaction for nitrite quantitation (**A**–**C**), while transcript levels were measured by real-time RT-PCR in cell extracts (**D**,**E**). (**A-C**): IL-6 and IL-1β were higher in Se Subopt supernatants [main diet effect: F (1,8) = 51.08 *p* < 0.0001 for IL-6, and F (1, 9) = 18.70 ^§§^*p* = 0.0019 for IL-1β]. 2-way ANOVA revealed a significant diet x Pb interaction for IL-6 [F (1, 10) = 6.869 *p* = 0.0256] and nitrites [F (1, 9) = 7.539 *p* = 0.0226], with significantly higher levels of these metabolites reached in Subopt/Pb OHSCs. Tukey’s post hoc analyses in (**A**–**C**) *p < 0.05, **p < 0.005, ***p < 0.0005. Mean ± SEM; n = 4 per group. (**D**,**E**): Arg-1 mRNA levels were higher in Subopt OHSCs compared to Opt [main diet effect F (1, 9) = 6.038 ^§^*p* = 0.0363]. In OHSCs from Pb exposed rats, Arg1 mRNA levels showed a tendency to decrease in both dietary groups [main Pb effect: F (1, 9) = 3.929 *p* = 0.0788]. CD11b levels were comparable in all groups. Mean ± SEM; n = 4 per group.

We then analyzed the reactivity to LPS of OHSCs from the different experimental groups, measuring the levels of IL-6, IL-1β, and nitrite in the culture media, following 24 h of stimulation (Fig. 6). An interaction between diet and Pb was found for IL-6 and nitrite that were increased by Pb in the Subopt condition (Fig. 6A,C). IL-1β protein levels (Fig. 6B) were higher in Subopt cultures as a whole, and not modified by Pb exposure (2-way ANOVA and Tukey’s post hoc analyses are reported in the figure legend).

RT PCR analysis of the LPS-induced expression of Arg-1 mRNA (Fig. 6D), showed significantly higher levels in the Se Subopt group as a whole compared to the Opt one. In OHSCs from Pb exposed rats, Arg1 mRNA levels showed a tendency to decrease in both dietary groups. CD11b mRNA expression, analyzed in the same cultures, remained unmodified in all groups (Fig. 6E).

## Discussion

In this study, we identified for the first time that maternal Se dietary restriction, developmental exposure to a low Pb dose, and their combination induce specific molecular changes in the offspring’s microglia evident from birth, thus advancing our knowledge of the cellular targets that may be responsible for the adverse neurobehavioral outcomes related to this condition, recently described by our group^30^.

First, we show that Se suboptimal diet, per se, increased whole brain levels of the inflammatory cytokines IL-6, IL-1β, and IL-10 and of the microglial phagocytic receptors CD11b and MRC-1 in newborn rats.

In cortical offspring microglia, we confirmed the higher expression of IL-6, IL-10, MRC-1, and a moderate increase of IL-1β and CD11b, in suboptimal Se condition supporting the acquisition of an inflammatory activation state that can influence fundamental physiological brain functions. Moreover, higher mRNA levels of iNOS and reduced levels of Arg-1 were found in suboptimal Se, an effect that was masked when analysing the whole brain.

Higher mRNA levels of IL-6, IL-1β, IL-10, and iNOS were observed also in organotypic hippocampal slices (OHSCs), suggesting a persistent inflammatory polarization occurring in the hippocampus at a more advanced stage of development. Indeed, the maturational stage reached by OHSCs at the time of stimulation (1 week post explant) roughly corresponds to PND 14–15, as hippocampal slices in vitro closely recapitulate the developmental program observed in vivo^46,47^.

The inflammatory molecules analyzed in this study play critical roles in almost every aspect of neural development, including neurogenesis, synaptogenesis, pruning and plasticity^48–50^. Dysregulations of cytokines, such as IL-1β, IL-6 and IL-10, and relative signaling pathways, have been implicated as factors involved in neurodevelopmental disorders^51^.

As an example, IL-1β and IL-6 has been found increased in maternal serum, amniotic fluid, and serum of children subsequently diagnosed with autism spectrum disorders (ASD) as well as in the well-known maternal immune activation rodent model^52^. A deregulated expression of the enzymes iNOS and Arg-1, which are mainly produced by microglia/macrophages in the CNS, may compromise important aspects of brain development^53,54^.

The microglial/macrophage-specific marker CD11b likewise displays a key developmental role, mediating the phagocytosis of supernumerary synapses and dendritic spines in the immature brain, thus contributing to neural circuit shaping. An altered complement signaling early in development results in sustained defects in synaptic connectivity, and it might contribute to neurodevelopmental diseases^55,56^. An important developmental function has been suggested also for the other microglial receptor analyzed, MRC-1^57^. Its deregulated expression was associated with abnormal neuronal development in a model of perinatal inflammation^58^.

Based on this evidence, we hypothesize that early microglial alterations described here can contribute, at least in part, to behavioral alterations later in life, as those reported in vivo in the same model of maternal Se restriction, including increased ultrasonic vocalisation emissions (an index of distress and increased emotional arousal), increased wall climbing, and locomotion^30^.

In addition, we provide evidence that optimal Se nutrition protects cortical microglia from the effects of Pb exposure, while some inflammatory changes are induced by Pb in hippocampal organotypic cultures also in optimal Se condition. The heterogeneity of the cell populations that constitute the OHSC model, which includes glial and neuronal cells, could in part account for the different response compared to isolated microglial cultures. In OHSCs, where the complex three-dimensional cell architecture is largely maintained, the basal behavior as well as the response to an inflammatory challenge are indeed the result of the crosstalk among the cell components, which contribute to the overall modulation of gene expression. Among the OHSC glial populations, astrocytes—playing along with microglia a key role in maintaining brain homeostasis and innate immune functions—are likely the main contributors to the different responses of OHSCs vs. primary microglia, through their activation and release of cytokines and other inflammatory mediators.

Beyond the mentioned differences between the two culture models, their responses are also likely reflecting the different susceptibility of cortex and hippocampus to Pb toxicity and to the effects of Pb interaction with Se status^59,60^. As we previously showed in vivo, optimal Se status during pregnancy counteracted the effects of Pb on somatic growth in pre-weaned pups and on locomotor activity and anxiety at later life stages, while Pb, regardless of Se status, altered glutamatergic receptor subunit trafficking to the post-synapse in the juvenile hippocampus and affected the long-term recognition memory in adult rats^30^.

Although the molecular mechanisms underlying Pb neurotoxicity as well as the protective effects of optimal Se need to be further explored, our study suggest the convergence of Pb and Se on common neuro-inflammatory pathways. While few papers reported pro-inflammatory effects of high-level of Pb exposure in the young rodent brain^61,62^, and several studies demonstrated Se antioxidant and anti-inflammatory effects^9–11,63^, we provide the first evidence of a possible interaction of maternal dietary Se and low-Pb exposure in the regulation of neuroplasticity- and neuroinflammatory-related genes in the offspring’s brain.

Another relevant aspect of our study is the observation that suboptimal Se nutrition, per se, and the combination with Pb exposure prime the developing brain enhancing its reactivity to a subsequent immune challenge, such as LPS. Indeed, both cortical microglia and hippocampal organotypic cultures from Se suboptimal fed-offspring showed a stronger response to LPS than optimal cultures, as indicated by higher levels of IL-6, IL1-β and—in the case of microglia—also of nitrite. The co-occurrence of suboptimal Se diet and Pb exposure exacerbated the LPS-induced production of nitrite, and increased that of CD11b in cortical microglia, as well as increased that of nitrite and IL-6 in OHSCs. These data reveal that the interaction among maternal dietary factors and an adverse environmental exposure since early phases of development set mechanisms of innate immune memory, inducing a long-lasting molecular reprogramming that potentially leads to harmful outcomes for the offspring facing a secondary insult.

This so-called immune “training”, to use a recently proposed nomenclature^64^, was formerly described in experimental and clinical conditions of neurodegenerative diseases. More recently, similarly to our findings, it was proposed as a mechanism that can be set also in the foetal life as a consequence of maternal stress^65^, maternal immune activation^66^, and maternal high-fat diet^38^, and that was associated to altered trajectories of brain maturation and increased vulnerability to psychopathologies in the offspring^67^.

The sexual dimorphism of microglia reactivity is increasingly viewed as a key factor determining the sex bias observed in many neurodevelopmental and neurological disorders, with males particularly vulnerable to life-long illnesses of neurodevelopmental origin. In this study, we analysed data by combining the results of the two sexes and this could have masked sex-specific effects, which deserve to be explored in future studies.

Despite this limitation, we believe that our results are highly informative on the importance of adequate maternal nutrition for the correct programming of offspring development and protection against neurotoxic substances.

Further studies are needed to understand the influence of cellular crosstalk, by mean of experimental cell co-culture systems, to address the molecular mechanisms underpinning offspring’s microglial training by low maternal Se intake and developmental Pb exposure, and to establish to which extent are these effects reversible and amenable of intervention.

## Conclusions

Overall, our findings support the notion that optimal maternal Se nutrition during pregnancy and lactation is essential for proper offspring development and that is able to counteract, at least in part, the toxicity of developmental exposure to Pb. These results also show that the interaction among maternal dietary factors and an adverse developmental environment can shape brain immune reactivity since early phases of development in a region-specific manner, possibly contributing to the risk of neurodevelopmental and neurodegenerative disorders.

## Data Availability

The datasets used and/or analyzed during the current study are available from the corresponding author on reasonable request.

## Abbreviations

Se: Selenium
Opt: Optimal
Subopt: Suboptimal
Pb: Lead
Veh: Vehicle
LPS: Lipopolysaccharide
CNS: Central nervous system
PND: Postnatal day
OHSC: Organotypic hippocampal slice cultures
IL-1β: Interleukin-1β
IL-6: Interleukin-6
IL-10: Interleukin-10
CD11b: Cluster of differentiation 11b
MRC-1: Mannose Receptor C-Type 1
iNOS: Nitric oxide synthase
Arg-1: Arginase-1

## References

[1] Barker DJP, Godfrey KM, Gluckman PD, Harding JE, Owens JA, Robinson JS (1993). Fetal nutrition and cardiovascular disease in adult life. Lancet.

[2] Barker DJ (1998). In utero programming of chronic disease. Clin. Sci. Lond. Engl. 1979.

[3] Schaefers ATU, Teuchert-Noodt G (2013). Developmental neuroplasticity and the origin of neurodegenerative diseases. World J. Biol. Psychiatry.

[4] Faa G, Manchia M, Pintus R, Gerosa C, Marcialis MA, Fanos V (2016). Fetal programming of neuropsychiatric disorders: Fetal programming. Birth Defects Res. Part C Embryo Today Rev..

[5] Rayman MP (2012). Selenium and human health. Lancet.

[6] Scientific opinion on dietary reference values for selenium. *EFSA J.* **12**(10), 3846 10.2903/j.efsa.2014.3846 (2014). 10.2903/j.efsa.2014.3846 PMC13136995 42087966

[7] Ojeda ML, Nogales F, Membrilla A, Carreras O (2019). Maternal selenium status is profoundly involved in metabolic fetal programming by modulating insulin resistance, oxidative balance and energy homeostasis. Eur. J. Nutr..

[8] Pappas AC, Zoidis E, Chadio SE (2019). Maternal selenium and developmental programming. Antioxidants.

[9] Beckett GJ, Arthur JR (2005). Selenium and endocrine systems. J. Endocrinol..

[10] Avery J, Hoffmann P (2018). Selenium, selenoproteins, and immunity. Nutrients.

[11] Fairweather-Tait SJ, Filippini T, Vinceti M (2022). Selenium status and immunity. Proc. Nutr. Soc..

[12] Zhang Y (2008). Comparative analysis of selenocysteine machinery and selenoproteome gene expression in mouse brain identifies neurons as key functional sites of selenium in mammals. J. Biol. Chem..

[13] Schweizer U, Fabiano M (2022). Selenoproteins in brain development and function. Free Radic. Biol. Med..

[14] Filipowicz D (2022). Selenium status and supplementation effects in pregnancy—A study on mother-child pairs from a single-center cohort. Nutrients.

[15] Skröder HM, Hamadani JD, Tofail F, Persson LÅ, Vahter ME, Kippler MJ (2015). Selenium status in pregnancy influences children’s cognitive function at 1.5 years of age. Clin. Nutr..

[16] Polanska K (2016). Selenium status during pregnancy and child psychomotor development—Polish Mother and Child Cohort study. Pediatr. Res..

[17] Polanska K (2017). Micronutrients during pregnancy and child psychomotor development: Opposite effects of zinc and selenium. Environ. Res..

[18] Shreenath, A. P., Ameer, M. A. & Dooley, J. Selenium deficiency. In *StatPearls*. (StatPearls Publishing, 2022) (Accessed 12 Dec 2022) http://www.ncbi.nlm.nih.gov/books/NBK482260/. 29489289

[19] Jones GD (2017). Selenium deficiency risk predicted to increase under future climate change. Proc. Natl. Acad. Sci..

[20] Stuss M, Michalska-Kasiczak M, Sewerynek E (2017). The role of selenium in thyroid gland pathophysiology. Endokrynol. Pol..

[21] Ajmone-Cat MA (2022). Critical role of maternal selenium nutrition in neurodevelopment: Effects on offspring behavior and neuroinflammatory profile. Nutrients.

[22] Canfield RL, Henderson CR, Cory-Slechta DA, Cox C, Jusko TA, Lanphear BP (2003). Intellectual impairment in children with blood lead concentrations below 10 μg per deciliter. N. Engl. J. Med..

[23] Wang M (2013). Effects of low-level organic selenium on lead-induced alterations in neural cell adhesion molecules. Brain Res..

[24] Latronico T (2022). Lead exposure of rats during and after pregnancy induces anti-myelin proteolytic activity: A potential mechanism for lead-induced neurotoxicity. Toxicology.

[25] Polanska K (2013). Developmental effects of exposures to environmental factors: The Polish mother and child cohort study. BioMed Res. Int..

[26] Dinçkol Ö, Fuentes B, Tartaglione AM, Pino A, Calamandrei G, Ricceri L (2022). Low-level lead exposure during development differentially affects neurobehavioral responses in male and female mouse offspring: A longitudinal study. NeuroToxicology.

[27] Bellinger DC, Chen A, Lanphear BP (2017). Establishing and achieving national goals for preventing lead toxicity and exposure in children. JAMA Pediatr..

[28] Hanna-Attisha M, Lanphear B, Landrigan P (2018). lead poisoning in the 21st century: The silent epidemic continues. Am. J. Public Health.

[29] EFSA Panel on Contaminants in the Food Chain (CONTAM) (2010). Scientific opinion on lead in food. EFSA J..

[30] Tartaglione AM (2022). Short- and long-term effects of suboptimal selenium intake and developmental lead exposure on behavior and hippocampal glutamate receptors in a rat model. Nutrients.

[31] Lu X, Zhang X, Li LY, Chen H (2014). Assessment of metals pollution and health risk in dust from nursery schools in Xi’an, China. Environ. Res..

[32] Garí M (2022). Prenatal exposure to neurotoxic metals and micronutrients and neurodevelopmental outcomes in early school age children from Poland. Environ. Res..

[33] Al-Saleh I, Al-Mohawes S, Al-Rouqi R, Elkhatib R (2019). Selenium status in lactating mothers-infants and its potential protective role against the neurotoxicity of methylmercury, lead, manganese, and DDT. Environ. Res..

[34] Liu M-C (2013). The effect of sodium selenite on lead induced cognitive dysfunction. NeuroToxicology.

[35] Li W-H, Shi Y-C, Tseng I-L, Liao VH-C (2013). Protective efficacy of selenite against lead-induced neurotoxicity in *Caenorhabditis elegans*. PLoS ONE.

[36] Bolton JL, Bilbo SD (2014). Developmental programming of brain and behavior by perinatal diet: Focus on inflammatory mechanisms. Dialogues Clin. Neurosci..

[37] Davis J, Mire E (2021). Maternal obesity and developmental programming of neuropsychiatric disorders: An inflammatory hypothesis. Brain Neurosci. Adv..

[38] Bordeleau M (2021). Maternal high-fat diet modifies myelin organization, microglial interactions, and results in social memory and sensorimotor gating deficits in adolescent mouse offspring. Brain Behav. Immun. Health.

[39] Desplats P, Gutierrez AM, Antonelli MC, Frasch MG (2020). Microglial memory of early life stress and inflammation: Susceptibility to neurodegeneration in adulthood. Neurosci. Biobehav. Rev..

[40] Lenz KM, Nelson LH (2018). Microglia and beyond: Innate immune cells as regulators of brain development and behavioral function. Front. Immunol..

[41] Meng X-L (2019). Selenoprotein SELENOK enhances the migration and phagocytosis of microglial cells by increasing the cytosolic free Ca^2+^ level resulted from the up-regulation of IP3R. Neuroscience.

[42] Huang Z, Rose AH, Hoffmann PR (2012). The role of selenium in inflammation and immunity: From molecular mechanisms to therapeutic opportunities. Antioxid. Redox Signal..

[43] Chibowska K (2020). Pre- and neonatal exposure to lead (Pb) induces neuroinflammation in the forebrain cortex, hippocampus and cerebellum of rat pups. Int. J. Mol. Sci..

[44] De Simone R (2013). Branched-chain amino acids influence the immune properties of microglial cells and their responsiveness to pro-inflammatory signals. Biochim. Biophys. Acta BBA Mol. Basis Dis..

[45] Antonietta Ajmone-Cat M, Mancini M, De Simone R, Cilli P, Minghetti L (2013). Microglial polarization and plasticity: Evidence from organotypic hippocampal slice cultures: Microglial Polarization and Tolerization. Glia.

[46] Muller D, Buchs P-A, Stoppini L (1993). Time course of synaptic development in hippocampal organotypic cultures. Dev. Brain Res..

[47] Simoni A, Griesinger CB, Edwards FA (2003). Development of rat CA1 neurones in acute versus organotypic slices: Role of experience in synaptic morphology and activity. J. Physiol..

[48] Bauer S, Kerr BJ, Patterson PH (2007). The neuropoietic cytokine family in development, plasticity, disease and injury. Nat. Rev. Neurosci..

[49] Deverman BE, Patterson PH (2009). Cytokines and CNS development. Neuron.

[50] Garay P (2010). Novel roles for immune molecules in neural development: Implications for neurodevelopmental disorders. Front. Synaptic Neurosci..

[51] Erbescu A, Papuc SM, Budisteanu M, Arghir A, Neagu M (2022). Re-emerging concepts of immune dysregulation in autism spectrum disorders. Front. Psychiatry.

[52] Ashwood P, Krakowiak P, Hertz-Picciotto I, Hansen R, Pessah I, Van de Water J (2011). Elevated plasma cytokines in autism spectrum disorders provide evidence of immune dysfunction and are associated with impaired behavioral outcome. Brain. Behav. Immun..

[53] Krystofova J, Pathipati P, Russ J, Sheldon A, Ferriero D (2018). The arginase pathway in neonatal brain hypoxia-ischemia. Dev. Neurosci..

[54] Tripathi MK, Kartawy M, Amal H (2020). The role of nitric oxide in brain disorders: Autism spectrum disorder and other psychiatric, neurological, and neurodegenerative disorders. Redox Biol..

[55] Brown GC, Neher JJ (2014). Microglial phagocytosis of live neurons. Nat. Rev. Neurosci..

[56] Druart M, Le Magueresse C (2019). Emerging roles of complement in psychiatric disorders. Front. Psychiatry.

[57] Burudi EME, Régnier-Vigouroux A (2001). Regional and cellular expression of the mannose receptor in the post-natal developing mouse brain. Cell Tissue Res..

[58] Pang Y (2016). Early postnatal lipopolysaccharide exposure leads to enhanced neurogenesis and impaired communicative functions in rats. PLoS ONE.

[59] Du Y (2015). ‘Chronic lead exposure and mixed factors of gender×age×brain regions interactions on dendrite growth, spine maturity and NDR kinase. PLoS ONE.

[60] Schneider JS, Anderson DW, Kidd SK, Sobolewski M, Cory-Slechta DA (2016). Sex-dependent effects of lead and prenatal stress on post-translational histone modifications in frontal cortex and hippocampus in the early postnatal brain. NeuroToxicology.

[61] Strużyńska L, Dąbrowska-Bouta B, Koza K, Sulkowski G (2007). Inflammation-like glial response in lead-exposed immature rat brain. Toxicol. Sci..

[62] Zhu J (2022). NLRP3 activation in microglia contributes to learning and memory impairment induced by chronic lead exposure in mice. Toxicol. Sci..

[63] Wu H (2022). Supplementation with selenium attenuates autism-like behaviors and improves oxidative stress, inflammation and related gene expression in an autism disease model. J. Nutr. Biochem..

[64] Neher JJ, Cunningham C (2019). Priming microglia for innate immune memory in the brain. Trends Immunol..

[65] Gómez-González B, Escobar A (2010). Prenatal stress alters microglial development and distribution in postnatal rat brain. Acta Neuropathol..

[66] Meyer U (2014). Prenatal poly(I:C) exposure and other developmental immune activation models in rodent systems. Biol. Psychiatry.

[67] Bordeleau M, Fernández de Cossío L, Chakravarty MM, Tremblay M-È (2021). From maternal diet to neurodevelopmental disorders: A story of neuroinflammation. Front. Cell. Neurosci..

